# Can circulating oxidative stress-related biomarkers be used as an early prognostic marker for COVID-19?

**DOI:** 10.3389/fmed.2023.1041115

**Published:** 2023-02-09

**Authors:** Pelin Uysal, Arzu Yüksel, Sinem Durmus, Çaglar Cuhadaroglu, Remise Gelisgen, Hafize Uzun

**Affiliations:** ^1^Department of Chest Diseases, Acibadem Mehmet Ali Aydinlar University Faculty of Medicine, Maslak Hospital, Istanbul, Turkey; ^2^Department of Biochemistry, Acibadem Mehmet Ali Aydinlar University Faculty of Medicine, Atakent Hospital, Istanbul, Turkey; ^3^Department of Medical Biochemistry, Cerrahpasa Faculty of Medicine, Istanbul University-Cerrahpasa, Istanbul, Turkey; ^4^Department of Chest Diseases, Acibadem University Faculty of Medicine, Altunizade Hospital, Istanbul, Turkey; ^5^Department of Medical Biochemistry, Faculty of Medicine, İstanbul Atlas University, Istanbul, Turkey

**Keywords:** COVID-19, nuclear factor kappa B, lipid metabolism, oxidized LDL, lectin-like oxidized-LDL receptor-1

## Abstract

**Background:**

Oxidative stress plays an important role in the pathogenesis of many diseases. This study aimed to investigate the relationship between nuclear factor kappa B (NF-κB) and oxidative stress and the severity of the disease in new COVID-19 patients, and, to compare the levels of NF-κB, oxidized LDL (oxLDL), and lectin-like oxidized-LDL receptor-1 (LOX-1) with oxygen saturation, which is an indicator of the severity parameters of the disease in COVID-19 patients.

**Methods:**

In this prospective study, 100 COVID-19 patients and 100 healthy subjects were selected.

**Results:**

LOX-1, NF-κB, and oxLDL were found to be higher in COVID-19 patients compared to the healthy subjects (*p* < 0.001 for all). According to the results of correlation analysis, it was found that there was no significant relationship between oxygen saturation and LOX-1, NF-κB and oxLDL parameters. There was significant relationship between oxLDL with LOX-1 and NF-κB in patients with COVID-19 disease. ROC analysis results of the highest discrimination power were oxLDL (AUC: 0.955, CI: 0.904–1.000; sensitivity: 77%, and specificity: 100%, for cutoff: 127.944 ng/l) indicating COVID-19.

**Conclusion:**

Oxidative stress plays an essential role in COVID-19. NF-κB, oxLDL, and LOX-1 seem to represent good markers in COVID-19. Our study also showed that oxLDL has the highest power in distinguishing patients with COVID-19 from the healthy subjects.

## Introduction

The virus, which emerged in late 2019 and called SARS-CoV-2 by the World Health Organization, spread rapidly around the world, causing a pandemic. While the disease called corona virus disease 2019 (COVID-19) progressed with asymptomatic or mild symptoms in some people, severe disease findings requiring hospitalization were observed in approximately 20% of the cases. The morbidity and mortality of COVID-19 is mostly associated with acute viral pneumonia and associated severe acute respiratory distress syndrome (ARDS) (1, 2).

Oxidative stress and inflammation cause oxidation of lipids and lipoproteins, and play an important role in the pathogenesis of many diseases (3). Oxidized LDL (oxLDL) formation is seen in various diseases resulting in oxidative stress and the presence of reactive oxygen species (ROS) (4). OxLDL leads to the progression of lesions in atherosclerosis, diabetes mellitus (DM), aging, non-alcoholic fatty liver, metabolic syndrome (MS), cardiovascular diseases (CVD) and cerebrovascular diseases, nephrotic syndrome, chronic renal failure, diabetic nephropathy, nephrosclerosis, and acute renal failure. It causes and plays an important role in the pathogenesis of the disease (5–7). It is expressed by pro-inflammatory and pro-oxidative stress in lectin-like oxidized-LDL receptor-1 (LOX-1) cells and endothelium, monocyte, macrophage, smooth muscle cells, cardiomyocytes, fibroblast, adipocyte, airway epithelial cells, dendritic cells, and thrombocytes (8–10).

LOX-1 expression increased by oxLDL stimulates the growth and proliferation of smooth muscle cells (SMC) with nuclear factor kappa B (NF-κB) and JNK signaling pathways, causing SMCs to move from the media to the subendothelial area. LOX-1 plays an important role in fibroblast growth and collagen synthesis stimulated by angiotensin II and transforming growth factor beta (TGF-β). The presence of LOX-1 in activated platelets is associated with thrombus formation (7).

In this respect, our aim in this study is to investigate the relationship between NF-κB and oxidative stress and the severity of the disease in new COVID-19 patients, and, to compare the levels of NF-κB, oxLDL, and LOX-1 with oxygen saturation (SO_2_), which is an indicator of the severity parameters of the disease in COVID-19 patients.

## Materials and methods

The study was designed prospectively. All procedures in our study comply with the 1964 Declaration of Helsinki and any subsequent amendments or comparable ethical standards. The study was approved by the Mehmet Ali Aydinlar University Faculty of Medicine Ethics Committee and informed consent was obtained from all participants (ATADEK-2020/17).

Of two hundred subjects who were admitted to Atakent Hospital emergency department between 15 March 2020 and 15 November 2022, 100 subjects with COVID-19 were included in this study. The recommended criteria established by the Scientific Committee of the Ministry of Health were used for the selection of possible COVID-19 patients. These criteria are having at least one sign or symptom of fever or acute respiratory illness (cough and respiratory distress); the presence of clinical features that cannot be explained by any other disease; the patient or a relative has a history of traveling to another country for 14 days prior to the onset of symptoms; and close contact with a patient confirmed to be COVID-19 positive by reverse transcription polymerase chain reaction (RT-PCR). Their diagnoses were confirmed by radiologist. The patients were selected from those over the age of 18 who applied to Acibadem Atakent Hospital with positive COVID-19 PCR and/or Thorax CT (100 cases) and 100 healthy subjects. Studies show that thorax CT has higher sensitivity than RT-PCR. RT-PCR false negatives can be quite high due to the immature development of nucleic acid detection technology, differences in detection rate from different manufacturers, low patient viral load, or inappropriate clinical sampling (11). In our study, 33 patients (33%) had positive RT-PCR results, 67 patients (67%) had negative RT-PCR results, and thorax CT results of all 89 patients were typically compatible with COVID-19 pneumonia. Patients were classified as uncomplicated, mild–moderate pneumonia, and severe pneumonia. Patients with symptoms such as fever, muscle/joint pain, cough, and sore throat, without respiratory distress (respiratory rate <24 per minute, SpO2 >93% in room air) and with normal chest X-ray and/or lung tomography were classified as uncomplicated. Patients with symptoms such as fever, muscle/joint pain, cough and sore throat, respiratory rate <30/min, SpO2 >90 in room air, and mild–moderate pneumonia findings on chest X-ray or tomography were classified as mild–moderate pneumonia. Patients with symptoms such as fever, muscle/joint pains, cough, and sore throat, with tachypnea (≥30/min), with SpO2 level below ≤90% in room air, and bilateral diffuse pneumonia findings on chest X-ray or tomography were classified as severe pneumonia. Control subjects were selected from individuals who were confirmed by PCR and clinical evaluation as not having COVID-19 disease and had no additional disease. All patients were of Turkish descent.

### Inclusion criteria

Age of COVID-19 diagnosis: ≥18 years. Patients who presented with positive SARS-CoV-2 RNA finding in throat swab samples and were diagnosed with COVID-19 according to the World Health Organization guidelines or having positive RT-PCR test with oxygen saturation rate below 90%.

### Exclusion criteria

Patients aged <18 years, cancer, obesity, diabetes, smoking, pregnant, and still hospitalized but not yet discharged from the hospital were excluded from this study.

After being diagnosed with COVID-19, treatment was given to all patients in line with the recommendations of the Scientific Board of the Turkish Ministry of Health. All patients were started on hydroxychloroquine loading 2*400 followed by 2 × 200 mg for a total of 5 days/azithromycin (500 mg on the first day followed by 250 for a total of 5 days) plus oseltamivir 75 mg given twice. Favipiravir was reserved for severe cases with dyspnea and SpO2 <90%. It was given in the dose of 1,600 mg twice on day one followed by 600 mg for the next 4 days.

The chest CT examinations were reported in accordance with the Radiological Society of North America (RSNA) Consensus Statement on Reporting Chest CT Findings Related to COVID-19.

Combined pharyngeal and nasopharyngeal swab samples were obtained for RT-PCR assay.

Samples were drawn into plain tubes with no additive, in the morning after an overnight fasting (10–12 h) from patients with COVID-19 and their respective controls. Serum samples were separated from cells immediately after centrifugation (4,000 rpm, 10 min at 4°C), aliquoted, and stored at −80°C until assayed for NF-κB, oxLDL, and LOX-1.

Routine biochemical parameters were measured by the autoanalyzer (Siemens Dimension, Germany). Serum C-reactive protein (CRP) levels were measured by a photometric method (Siemens Dimension, Germany). Complete blood count parameters were obtained with automatic hematology analyzer (Sysmex XN-2000, Germany).

Oxygen saturation (SO_2_) levels were measured by blood gas analyze (Siemens Rapid 500, Germany).

### Measurement of serum NF-κB p65 levels

Serum NF-κB levels were measured by a commercially available competitive enzyme-linked immunoassay kit (R&D Systems, Minneapolis, MN, United States). The coefficients of intra- and inter-assay variations were 4.6% (*n* = 20) and 5.4% (*n* = 20), respectively.

### Measurement of serum oxidized low density lipoprotein (oxLDL) levels

Serum oxLDL levels were measured by a commercially available competitive enzyme linked immunoassay kit (R&D Systems, Minneapolis, MN, United States). The coefficients of intra- and inter-assay variation were 4.5% (*n* = 20) and 5.3% (*n* = 20), respectively.

### Measurement of serum lectin-like oxidized-low density lipoprotein receptor 1 (LOX-1) levels

Serum LOX-1 levels were measured by a commercially available competitive enzyme linked immunoassay kit (R&D Systems, Minneapolis, MN, United States). The coefficients of intra- and inter-assay variation were 4.7% (*n* = 20) and 5.8% (*n* = 20), respectively.

### Statistical analysis

In the statistical analysis of the study, the criteria discussed were defined by mean, standard deviation, frequency, and percentage values. The distribution of all analyzed parameters was confirmed using the Kolmogorov–Smirnov test. Results for normally distributed continuous variables were expressed as means ± standard deviations. Chi-Square test was used to compare frequencies and percentages between the groups. Student’s *t*-test was used for comparisons of two groups. One-way ANOVA and Tukey test as *post-hoc* were used in the comparison of three groups. Correlation analysis was performed using Spearman’s correlation analysis. In the study cases, receiver operating characteristic (ROC) curve analysis was used to investigate the performance of important criteria in determining disease diagnosis. As a result of the ROC analysis, cutoff points were determined by using the Youden Index. A *p* value below 0.05 was expressed as significant. *Post-hoc* power analysis was performed for the study, and the power of the study was found to be 100% at α = 0.05, for all three parameters (LOX-1, NF-κB, and oxLDL). All statistical analyses were carried out using Statistical Product and Service Solutions (SPSS) v. 21.0 (IBM, Armonk, NY, United States) package program.

## Results

**Figure 1:**
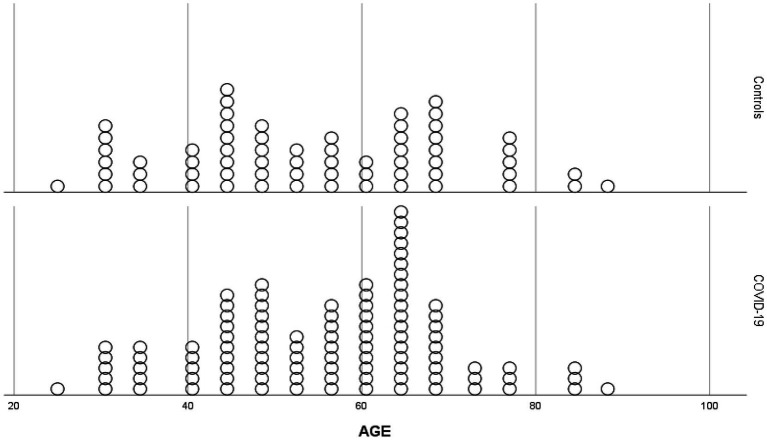
Dot graphs of population age distribution.

Both groups were selected from patients and healthy subjects matched for gender and age (no statistically significant difference). Dot graph of population age distribution was also shown in Figure 1. When the clinical results of the patients were evaluated, no significant difference was found between diastolic blood pressure (DBP), systolic blood pressure (SBP), and SO_2_ levels. However, PCR and radiological findings were found to be significantly positive in patients with COVID-19 (both *p* < 0.001). No significant difference was found between the groups in terms of survive status (Table 1). Clinical parameters of the COVID-19 patients are also shown in Table 2.

**Table 1:** Demographic data and clinical data results at admission to hospital.

Parameters	COVID-19 (*n* = 100)	Healthy subjects (*n* = 100)	*p*
Gender female/male (*n*, %)	32 (32%) / 68 (68%)	31 (31%) /69 (69%)	0.879
Age (year) (mean ± SD)	55.96 ± 13.74	54.50 ± 15.68	0.138
DBP (mmHg) (mean ± SD)	71.74 ± 13.51	77.08 ± 13.23	0.692
SBP (mmHg) (mean ± SD)	127.34 ± 28.60	132.67 ± 34.06	0.120
SO_2_ (%) (mean ± SD)	91.52 ± 9.18	92.65 ± 5.67	0.594
PCR results negative/positive (*n*, %)	67 (67%) / 33 (33%)	100 (100%)/0 (0%)	**0.000**
Radiological findings negative/positive (*n*, %)	0 (0%)/100 (100%)	100 (100%)/0 (0%)	**0.000**
Survive status Ex/healed (*n*, %)	5 (5%)/95 (95%)	0 (0%)/100 (100%)	0.066

Categorical data (gender, PCR results, radiological findings, survive status) were analyzed with the Chi-square test. Student’s *t*-test was used to compare the means between the two groups (COVID-19 and healthy subjects) for other parameters (age, SBP, and SO2). Bold *p* values express statistically significant.

**Table 2:** Clinical parameters of the COVID-19 patients.

	Value	Range
Duration of disease, days	8	2–15
Fever, *n* (%)	84 (84)	
Cough, *n* (%)	79 (79)	
Loss of smell and taste, *n* (%)	17 (17)	
Dyspnea, *n* (%)	55 (55)	
Respiratory distress, *n* (%)	26 (26)	
Diarrhea, *n* (%)	5 (5)	
Common body pain	14 (14)	
Myalgia, *n* (%)	8 (8)	
Headache, *n* (%)	7 (7)	

Hematological values and routine biochemical tests of the patients were evaluated, and no significant difference was found (Tables 3, 4). There was no significant difference in total cholesterol, high density lipoprotein (HDL), LDL, and triglyceride levels between the groups (*p* > 0.05 for all; Table 4). All three parameters LOX-1, NF-κB, and ox-LDL were found to be higher in COVID-19 patients compared to the healthy subjects (*p* < 0.001 for all) (Table 5). There was significant relationship between oxLDL with LOX-1 (*r*: 0.921; *p* < 0.0001) and NF-κB (*r*: 0.712; *p* < 0.0001) in patients with COVID-19 disease. The TC scanning of these patients was evaluated and no correlation was found between the change in TC and biomarker levels.

**Table 3:** Hematological parameters of the subjects included in examined groups.

Parameters	COVID-19 (mean ± SD)	Healthy subjects (mean ± SD)	*p*
RBC (×10^6^/μL)	4.31 ± 0.80	3.93 ± 0.82	0.923
HGB (g/dL)	12.03 ± 2.15	11.06 ± 2.15	0.724
Hct (%)	36.84 ± 5.68	35.09 ± 5.97	0.794
PLT (×10^4^/μL)	216.41 ± 114.90	197.95 ± 110.86	0.588
Neut (×10^9^/L)	68.63 ± 20.22	70.68 ± 18.93	0.636
Lymph (×10^9^/L)	19.47 ± 13.92	19.88 ± 16.82	0.213
MONO (x10^3^/μL)	8.61 ± 5.09	8.21 ± 6.41	0.186
EOS (x10^3^/μL)	1.04 ± 2.45	0.87 ± 1.24	0.377
ESR (mm/h)	15.55 ± 7.21	13.46 ± 4.45	0.730

RBC, red blood cell; HGB, hemoglobin; Hct, hematocrit; PLT, platelet; Neut, neutrophil; Lymph, lymphocyte; MONO: monocyte; EOS: eosinophil; ESR: erythrocyte sedimentation rate. Student’s *t*-test was used to compare the means between the two groups (COVID-19 and healthy subjects) for all parameters.

**Table 4:** Biochemical parameters of the subjects included in the study.

Parameters	COVID-19 (mean ± SD)	Healthy subjects (mean ± SD)	*p*
Glucose (mg/dL)	138.45 ± 92.67	135.35 ± 78.89	0.739
Urea (mg/dL)	52.83 ± 42.49	42.83 ± 30.45	0.356
Creatinine (mg/dL)	1.45 ± 1.45	1.23 ± 0.37	0.794
Total cholesterol (mg/dL)	164.35 ± 33.45	154.73 ± 35.63	0.346
HDL (mg/dL)	59.54 ± 12.54	64.45 ± 13.23	0.468
LDL (mg/dL)	122.58 ± 22.34	120.34 ± 16.23	0.677
Triglyceride (mg/dL)	106.6 ± 22.54	102.24 ± 24.45	0.455
Total protein (g/dL)	7.02 ± 0.89	7.23 ± 3.25	0.623
Albumin (g/dL)	3.12 ± 0.37	3.14 ± 0.67	0.938
T. bilirubin (mg/dL)	0.74 ± 0.81	0.80 ± 0.73	0.603
D. bilirubin (mg/dL)	0.34 ± 0.52	0.32 ± 0.42	0.893
CRP (mg/L)	7.54 ± 6.89	6.63 ± 6.12	0.235
Na (mmol/L)	137.19 ± 4.74	136.45 ± 5.25	0.412
K (mmol/L)	4.35 ± 1.26	4.63 ± 0.73	0.473
Cl (mmol/L)	100.63 ± 5.29	101.83 ± 4.03	0.711
AST (U/L)	48.82 ± 41.86	45.92 ± 45.23	0.814
ALT (U/L)	45.38 ± 41.59	45.42 ± 41.23	0.729
LDH (U/L)	356.86 ± 193.64	351.54 ± 205.57	0.913
ALP (U/L)	157.79 ± 168.97	134.87 ± 146.46	0.529
GGT (U/L)	133.56 ± 94.89	85.46 ± 44.98	0.286
CK (U/L)	538.97 ± 125.56	163.98 ± 162.68	0.292
CK-MB (U/L)	1.67 ± 3.16	1.84 ± 1.48	0.793
Ferritin (ng/mL)	1303.06 ± 4882.87	685.02 ± 757.04	0.090
Troponin T (μg/L)	0.05 ± 0.11	0.06 ± 0.12	0.923
PT (s)	13.71 ± 2.25	13.72 ± 1.68	0.996
APTT (s)	25.28 ± 4.34	23.23 ± 3.80	0.334
Fibrinogen (mg/dL)	541 ± 132	525 ± 159	0.612
D-Dimer (mg/dL)	3.23 ± 6.23	3.01 ± 3.78	0.656
NT-ProBNP (pg/mL)	2388.85 ± 7843.63	3391.15 ± 7000.45	0.476
PCT (ng/mL)	0.21 ± 0.20	0.23 ± 0.01	0.878

ALT, alanine aminotransferase; AST, aspartate aminotransferase; CK, creatine kinase; CK-MB, creatine kinase-MB; LDH, lactate dehydrogenase; ALP, alkaline Phosphatase; GGT, gamma-glutamyl transferase; NT-Pro-BNP, N-terminal pro-Brain natriuretic peptide; Na, sodium; K, potassium; Cl, chlorine; CRP, C-reactive protein; PT, prothrombin time; APTT, activated partial thromboplastin time; HDL, high density lipoprotein; LDL, low density lipoprotein; PCT, procalcitonin. Student’s *t*-test was used to compare the means between the two groups (COVID-19 and healthy subjects) for other parameters.

**Table 5:** Oxidative stress-related biomarkers of the subjects included in the study.

	COVID-19 (mean ± SD)			Healthy subjects (mean ± SD)			*p*
	Age < 50	Age > 50	Total	Age < 50	Age > 50	Total	
LOX-1 (ng/mL)	3.70 ± 3.82	3.94 ± 3.86	3.86 ± 3.82	2.70 ± 1.43	3.16 ± 1.68	2.94 ± 1.56	**0.000**
NF-κB (pg/mL)	1244.57 ± 1699.47	1141.92 ± 1425.38	1176.94 ± 1515.07	564.78 ± 220.74	553.94 ± 222.28	559.15 ± 219.43	**0.000**
oxLDL (ng/L)	131.13 ± 12.20	133.16 ± 12.06	132.47 ± 12.07	121.23 ± 15.95	113.16 ± 20.15	117.04 ± 18.53	**0.000**

NF-κB, nuclear factor kappa B; oxLDL, oxidized LDL; LOX-1, lectin-like oxidized-LDL receptor-1. Student’s *t*-test was used to compare the means between the two groups (COVID-19 and healthy subjects) for other parameters. Bold text expresses statistically significant.

In the subgroups formed according to disease severity, LOX-1 levels in patients with severe pneumonia were found to be statistically significantly higher than both uncomplicated COVID-19 patients (*p* < 0.01) and mild–moderate pneumonia patients (*p* < 0.05). While oxLDL levels were found to be higher in patients with severe pneumonia than only uncomplicated COVID-19 patients (*p* < 0.05), no significant difference was found between NF-κB values according to disease severity. In Ex patients, LOX-1, NF-κB, and oxLDL levels were all found to be higher compared to the healed patients (*p* < 0.05 for all), (Table 6).

**Table 6:** Oxidative stress-related biomarkers of the subgroups according to severity grading and survival status.

Parameters groups	LOX-1 (ng/ml)	NF-κB (pg/ml)	oxLDL (ng/L)
**Severity grading**			
Uncomplicated (*n* = 25)	6.62 ± 2.72	900.16 ± 570.23	130.21 ± 16.35
Mild–moderate pneumonia (*n* = 55)	6.92 ± 1.75	983.92 ± 511.92	134.83 ± 11.12
Severe pneumonia (*n* = 20)	7.93 ± 0.96 a**, b*	1018.00 ± 935.60	138.10 ± 7.18 a*
**Survival status**			
Healed (*n* = 95)	5.33 ± 1.82	593.28 ± 144.82	116.71 ± 14.94
Ex (*n* = 5)	7.21 ± 1.56 c*	1007.86 ± 793.67 c*	133.61 ± 11.98 c*

Three subgroups were formed according to the disease severity rating. One-way ANOVA test was used to compare the three groups and Tukey test was used as *post-hoc* test. Two subgroups were formed for survival status and statistical analyzes were performed with Mann–Whitney test. a means versus uncomplicated; b means versus mild–moderate pneumonia; c means versus healed; *p < 0.05; **p < 0.01; ***p < 0.001.

**Figure 2:**
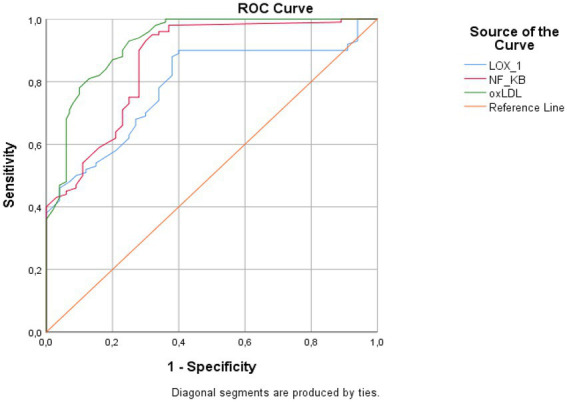
Diagnostic criteria of ROC curve for selected parameters in COVID-19.

ROC analysis results for important biochemical parameters showed that all three parameters (LOX-1, NF-κB, and oxLDL) had high discrimination power from patients with COVID-19 (Figure 2). Accordingly, LOX-1 was found to be the parameter with the lowest discrimination power (AUC: 0.787, CI: 0.722–0.852; sensitivity: 88%; and specificity: 62%, for cutoff: 1.93 ng/ml), while the parameter with the highest discrimination power was oxLDL (AUC: 0.26, CI: 0.892–0.961; sensitivity: 81%; and specificity: 87%, for cutoff: 124.38 ng/l) (Table 7).

**Table 7:** Cutoff values, sensitivity and specificity values by ROC analysis for LOX-1, NF-κB and ox-LDL values indicating COVID-19.

Variables	AUC	Std. error	*p*	Asymptotic 95% CI		Positive if greater than or equal to	Sensitivity	Specificity
				Lower bound	Upper bound			
LOX-1	0.787	0.033	0.000	0.722	0.852	2.116	88%	62%
NF-κB	0.864	0.025	0.000	0.815	0.914	476.26	97%	63%
oxLDL	0.926	0.018	0.000	0.892	0.961	124.38	81%	87%

NF-κB, nuclear factor kappa B; oxLDL, oxidized LDL; LOX-1, lectin-like oxidized-LDL receptor-1.

## Discussion

To the best of our knowledge, this is the first report to showing changes in three key parameters as NF-κB, oxLDL, and LOX-1 in systemic oxidative stress using an analysis of multiple biomarkers in COVID-19 patients. We believe that oxidative stress plays an important role in the pathological development of COVID-19.

The most important contributions of the laboratory findings are that they give an idea about the staging, prognosis, and therapeutic monitoring of COVID-19. Many laboratory tests can be very helpful in determining the severity of the disease and in determining the risk of development of ARDS, disseminated intravascular coagulation (DIC), and multiple organ failure (1, 2). Biochemical monitoring of COVID-19 patients with *in vitro* diagnostic tests is crucial for assessing disease diagnosis, severity, and progression, as well as monitoring therapeutic intervention. In addition to the laboratory tests commonly used in routine, new evidence suggests that serious COVID-19 patients may be at risk for cytokine storm syndrome. Defining laboratory tests that will contribute to the diagnosis and follow-up of COVID-19 patients is very important to distinguish between severe and non-severe cases, and to identify those with low or high mortality risk, in addition to the stage of diagnosis.

We examine the biochemical events that characterize the progression of COVID-19 in relation to three markers sensitive to increased ROS as possible candidates in the of NF-κB, oxLDL, and LOX-1. NF-κB is a transcription factor, oxLDL is a critical factor in atherogenesis, and LOX-1 is the major receptor for oxLDL in human endothelial cells.

Stimuli that increase LOX-1 expression are cytokines, interferon-V (IFN-V), oxLDL and other modified lipoproteins, free radicals, TNF-α, interleukin (IL-1β), TGF-β1, superoxide anion hydrogen peroxide (H_2_O_2_), 8-isoprostoglandin F2-α, and lysophosphatidylcholine (7, 12–14). Macrophages trained by oxLDL encounter large amounts of oxidized lipids in the infected areas and exhibit greater oxidized lipid uptake, leading to transient lipid depletion (8).

In COVID-19, thrombosis occurs because of blood clotting, causing blockages in the heart, lungs and vessels. Thrombotic events occur very frequently in intensive care patients for a variety of reasons. Chemokines (E, P selectin), intracellular adhesion molecule-1 (ICAM-1), and vascular cell adhesion molecule-1 (VCAM-1) by activation of NF-κB because of stimulation of vascular cells by binding oxLDL to LOX-1 lead to increased expression of granulocyte and macrophage colony-stimulating factors and monocyte chemotactic protein-1 (MCP1), leading to proatherogenic effects (7).

In SARS-CoV-infected macrophages, NF-κB is activated by the CARD9-BCL10 (Caspase recruitment domain family member 9-B-cell lymphoma/leukemia 10) module and triggers the major inflammatory adapter protein apoptosis-associated speck-like protein containing a caspase recruitment domains (ASC) (15). Reactive oxygen and nitrogen species are also a signaling molecule responsible for the release of pro-inflammatory cytokines. In particular, it activates redox-sensitive transcription factors such as NF-κB, which play a key role in inflammation (16). Oral and intravenous glutathione (GSH), GSH precursor N-acetyl-cysteine (NAC), and alpha lipoic acid (ALA) are known to block NF-κB. These molecules are new treatment approaches used for respiratory distress and cytokine storm syndrome in COVID-19 pneumonia (17). In current study, all three parameters LOX-1, NF-κB, and oxLDL were found to be higher in COVID-19 patients compared to the healthy subjects. Moreover, there were significant relationship between oxLDL with LOX-1 and NF-κB in patients with COVID-19 disease. At the same time, no significant difference was found between the groups in total-cholesterol, HDL-cholesterol, and LDL-cholesterol levels. Interestingly, Hu et al. (9) showed that serum lipid levels, especially total-cholesterol, HDL-cholesterol, and LDL-cholesterol, were significantly lower in patients with COVID-19 infection. In fact, up to 90% of circulating oxLDL can be found in oxLDL immune complexes (oxLICs). Preparation of OxLIC-mediated inflammatory signalosomes in macrophages is dependent on CARD9. It is a critical adapter protein and a central integrator in innate immune cell activation that triggers the inflammatory signaling pathway in response to viral infection. CARD9 signalosome, a triple protein complex consisting of CARD9, BCL10, and MALT1 (CBM complex), leads to the activation of NF-κB and mitogen activated protein kinase (MAPK) signaling result in up-regulation of several cytokines and chemokines (10). Wei et al. (18) reported that the development of hypolipidemia started in patients with mild symptoms and gradually worsened in relation to the severity of the disease. Acute inflammation caused by SARS-COV-2 alters lipid metabolism. Pro-inflammatory cytokines such as TNF-α, IL-6, and IL-1β have been reported to modulate lipid metabolism by altering liver function and reducing cholesterol influx and transport. It was reported that IL-6 increased dramatically in 96% of all patients. They demonstrated that pro-inflammatory cytokines such as IL-6 are a significant contributor to lipid abnormalities in patients. This strongly suggests that pro-inflammatory cytokines such as IL-6 are a significant contributor to lipid abnormalities in patients. Lipids are highly vulnerable to the degradation of free radicals, which are usually raised in host cells with a viral infection (19). Assignment of oxLDL in the serum of patients will be required to determine this possibility.

Pincemail et al. (20) have recently proven that systemic oxidative stress increases lipid peroxidation in critically ill COVID-19 patients, as well as deficiencies in some antioxidants (vitamin C, glutathione, and thiol proteins) and trace elements (selenium). Strong positive correlations between lipid peroxides and Cu and the negative correlation between γ-tocopherol and Cu highlighted the role played by copper in increased oxidative stress in COVID-19 patients. Oxidative stress is increased in patients with COVID-19 and is associated with mortality (21).

LOX-1 levels were found to be higher in patients with severe pneumonia compared to both uncomplicated COVID-19 patients and patients with mild to moderate pneumonia, while oxLDL levels were found to be higher in patients with severe pneumonia than in patients with only uncomplicated COVID-19. At the same time, in Ex patients, LOX-1, NF-κB, and oxLDL levels were all found to be higher compared to the healed patients. NF-κB, oxLDL, and LOX-1 levels are independent risk factors in determining the prognosis of COVID-19. COVID-19 is a disease whose activity is still very high and continues to spread rapidly. Scientists continue to search for markers that can help with the early detection and prognosis of this disease. The results of our study are consistent with studies reporting that oxidative stress markers are significantly increased in COVID-19 patients (22, 23). We think that the biochemical parameters mentioned in our study are important in determining the prognosis of the disease and may be beneficial, together with other risk factors, in terms of the need for transfer to the intensive care unit after the initial evaluation of the patients in the emergency unit (24–26).

In general, increased oxidative stress with all symptoms in patients diagnosed with COVID-19; may cause an increase in the symptoms of this chronic disease in individuals with other chronic diseases. This may cause the progression of COVID-19 in individuals to be severe and heavy. Accordingly, it has been predicted that supplementing diagnosed COVID-19 patients with strong antioxidant substances may have a protective effect. For example, ginger extract is a wealthy source of antioxidants and can be used to prevent the glycation and oxidative stress-induced damage of biomolecules in various health complications including inflammation (27). In many studies, the administration of various antioxidants as both prophylactic and therapeutic support to COVID-19 patients has been shown to reduce their symptoms (17, 28–47).

In the current study, 33 patients (33%) had positive RT-PCR results, 67 patients (67%) had negative RT-PCR results, and thorax CT results of all 89 patients were typically compatible with COVID-19 pneumonia. Combined pharyngeal and nasopharyngeal swab (RT-PCR) tests may be negative, especially in the early phase of the disease or when the viral load is low, and CT plays an important role in the diagnosis of COVID-19 during this period. The sensitivity of CT in the diagnosis of COVID-19 was found to be 98%, while the sensitivity of RT PCR, which is the gold standard diagnostic test, was found to be 71% in the early period (11). Although RT-PCR test is the gold standard for the diagnosis of COVID-19, chest X-ray and CT have an important role in the diagnosis, follow-up, and staging of pneumonia (48, 49).

Although our study has strengths, it has some limitations. However, as can be seen in the results we presented, we see that the presence or absence of each of these comorbid diseases does not cause a change in NF-κB, oxLDL, and LOX-1 levels, which are the main parameters of our study. The limitation of this study is the small number of the patients included in a single center. We used a convenience sample which may increase the risk of selection bias. Our study did not allow us to conclude whether increased oxidative stress could be directly attributed to COVID-19 disease.

NF-κB, oxLDL, and LOX-1 seem to represent good markers in COVID-19. Oxidative stress may play a critical role in the development of COVID-19 disease and of damage to the COVID-19 patients. Our study also showed that oxLDL has the highest power in distinguishing patients with COVID-19 from healthy subjects. Since increased oxidative stress was observed in patients who died compared to those who recovered, we think that the use of NF-κB, oxLDL, and LOX-1 levels at the onset of COVID-19 disease is a valuable starting point for the general assessment of oxidative stress, and thus provides better triage for patients in terms of disease severity. Antibiotics are produced and fought against bacteria, but the battle against viruses must be very different. At this point, the strongest weapon is the immune system. Because viruses undergo mutations and can appear in different masks. According to these results, it was predicted that supplementation with strong antioxidant substances may have a protective effect in diagnosed COVID-19 patients. The levels of these biomolecules can guide future patient-targeted therapies in clinical practice.

## Data availability statement

The datasets presented in this study can be found in online repositories. The names of the repository/repositories and accession number(s) can be found in the article/supplementary material.

## Ethics statement

The studies involving human participants were reviewed and approved by the study was approved by the Mehmet Ali Aydinlar University Faculty of Medicine Ethics Committee and informed consent was obtained from all participants (ATADEK 2020/17). The patients/participants provided their written informed consent to participate in this study.

## Author contributions

PU and HU participated in the design of the study and wrote the manuscript. PU and ÇC collected and determined the patients. AY, SD, RG, and HU carried out the ELISA test. SD performed the statistical analysis. All authors contributed to the article and approved the submitted version.

## Conflict of interest

The authors declare that the research was conducted in the absence of any commercial or financial relationships that could be construed as a potential conflict of interest.

## Publisher’s note

All claims expressed in this article are solely those of the authors and do not necessarily represent those of their affiliated organizations, or those of the publisher, the editors and the reviewers. Any product that may be evaluated in this article, or claim that may be made by its manufacturer, is not guaranteed or endorsed by the publisher.

## Abbreviations

ALA: Alpha lipoic acid
ARDS: Acute respiratory distress syndrome
ASC: Apoptosis-associated speck-like protein containing a caspase recruitment domains
CRP: C-reactive protein
CVD: Cardiovascular diseases
DBP: Diastolic blood pressure
DM: Diabetes mellitus
GSH: Glutathione
HDL: High density lipoprotein
ICAM-1: Intracellular adhesion molecule-1
IFN-V: Interferon-V
IL-1β: Interleukin-1 beta
LOX-1: Lectin-like oxidized-LDL receptor-1
MAPK: Mitogen activated protein kinase
MCP1: Monocyte chemotactic protein-1
MS: Metabolic syndrome
NAC: N-acetyl-cysteine
NF-κB: Nuclear factor kappa B
oxLDL: Oxidized low density lipoprotein
oxLIC: oxLDL immune complex
ROC: Receiver operating characteristic
ROS: Reactive oxygen species
RT-PCR: Reverse transcription polymerase chain reaction
SBP: Systolic blood pressure
SMC: Smooth muscle cells
SO_2_: Oxygen saturation
SPSS: Statistical product and service solutions
TGF-β: Transforming growth factor beta
VCAM-1: Vascular cell adhesion molecule-1.

## References

[1] Sarangi MK Padhi S Dheeman S Karn SK Patel LD Yi DK. Diagnosis, prevention, and treatment of coronavirus disease: a review. Expert Rev Anti-Infect Ther. (2022) 20:243–66. doi: 10.1080/14787210.2021.1944103 34151679

[2] Chen JM. Novel statistics predict the COVID-19 pandemic could terminate in 2022. J Med Virol. (2022) 94:2845–8. doi: 10.1002/jmv.27661, PMID: 35150458 PMC9088340

[3] Kim HJ Yuan J Norris K Vaziri ND. High-calorie diet partially ameliorates dysregulation of intrarenal lipid metabolism in remnant kidney. J Nutr Biochem. (2010) 21:999–1007. doi: 10.1016/j.jnutbio.2009.08.006, PMID: 19954950 PMC3206097

[4] Schreurs MP Cipolla MJ. Cerebrovascular dysfunction and blood-brain barrier permeability induced by oxidized LDL are prevented by apocynin and magnesium sulfate in female rats. J Cardiovasc Pharmacol. (2014) 63:33–9. doi: 10.1097/FJC.0000000000000021, PMID: 24084218 PMC3909873

[5] Oteiza A Li R McCuskey RS Smedsrød B Sørensen KK. Effects of oxidized low-density lipoproteins on the hepatic microvasculature. Am J Physiol Gastrointest Liver Physiol. (2011) 301:G684–93. doi: 10.1152/ajpgi.00347.2010, PMID: 21778464

[6] Maiolino G Rossitto G Caielli P Bisogni V Rossi GP Calò LA. The role of oxidized low-density lipoproteins in atherosclerosis: the myths and the facts. Mediat Inflamm. (2013) 2013:1–13. doi: 10.1155/2013/714653 PMC3816061 24222937

[7] Pirillo A Norata GD Catapano AL. LOX-1, OxLDL, and atherosclerosis. Mediat Inflamm. (2013) 2013:1–12. doi: 10.1155/2013/152786 PMC3723318 23935243

[8] Levitan I Shentu TP. Impact of oxLDL on cholesterol-rich membrane rafts. J Lipids. (2011) 2011:1–11. doi: 10.1155/2011/730209 PMC3066652 21490811

[9] Hu X Chen D Wu L He G Ye W. (2020). Low Serum Cholesterol Level among Patients with COVID-19 Infection in Wenzhou, China. Available at: https://ssrn.com/abstract=3544826 (Accessed June 10, 2022).

[10] Zhong X Chen B Yang L Yang Z. Molecular and physiological roles of the adaptor protein CARD9 in immunity. Cell Death Dis. (2018) 9:52. doi: 10.1038/s41419-017-0084-6, PMID: 29352133 PMC5833731

[11] Fang Y Zhang H Xie J Lin M Ying L Pang P. Sensitivity of chest CT for COVID-19: comparison to RT-PCR. Radiology. (2020) 296:E115–7. doi: 10.1148/radiol.2020200432, PMID: 32073353 PMC7233365

[12] Yoshimoto R Fujita Y Kakino A Iwamoto S Takaya T Sawamura T. The discovery of LOX-1, its ligands and clinical significance. Cardiovasc Drugs Ther. (2011) 25:379–91. doi: 10.1007/s10557-011-6324-6, PMID: 21805404 PMC3204104

[13] Xu S Ogura S Chen J Little PJ Moss J Liu P. LOX-1 in atherosclerosis: biological functions and pharmacological modifiers. Cell Mol Life Sci. (2013) 70:2859–72. doi: 10.1007/s00018-012-1194-z, PMID: 23124189 PMC4142049

[14] Navarra T Del Turco S Berti S Basta G. The lectin-like oxidized low-density lipoprotein receptor-1 and its soluble form: cardiovascular implications. J Atheroscler Thromb. (2010) 17:317–31. doi: 10.5551/jat.3228, PMID: 20009416

[15] Poeck H Bscheider M Gross O Finger K Roth S Rebsamen M. Recognition of RNA virus by RIG-I results in activation of CARD9 and inflammasome signaling for interleukin 1 beta production. Nat Immunol. (2010) 11:63–9. doi: 10.1038/ni.1824, PMID: 19915568

[16] DeDiego ML Nieto-Torres JL Regla-Nava JA Jimenez-Guardeño JM Fernandez-Delgado R Fett C. Inhibition of NF-κB-mediated inflammation in severe acute respiratory syndrome coronavirus-infected mice increases survival. J Virol. (2014) 88:913–24. doi: 10.1128/JVI.02576-13, PMID: 24198408 PMC3911641

[17] Horowitz RI Freeman PR Bruzzese J. Efficacy of glutathione therapy in relieving dyspnea associated with COVID-19 pneumonia: a report of 2 cases. Respir Med Case Rep. (2020) 30:101063. doi: 10.1016/j.rmcr.2020.101063, PMID: 32322478 PMC7172740

[18] Wei X Zeng W Su J Wan H Yu X Cao X. Hypolipidemia is associated with the severity of COVID-19. J Clin Lipidol. (2020) 14:297–304. doi: 10.1016/j.jacl.2020.04.008, PMID: 32430154 PMC7192140

[19] Zidar DA Juchnowski S Ferrari B Clagett B Pilch-Cooper HA Rose S. Oxidized LDL levels are increased in HIV infection and may drive monocyte activation. J Acquir Immune Defic Syndr. (2015) 69:154–60. doi: 10.1097/QAI.0000000000000566, PMID: 25647528 PMC4446174

[20] Pincemail J Cavalier E Charlier C Cheramy-Bien JP Brevers E Courtois A. Oxidative stress status in COVID-19 patients hospitalized in intensive care unit for severe pneumonia. A pilot study. Antioxidants (Basel). (2021) 10:257. doi: 10.3390/antiox10020257, PMID: 33562403 PMC7914603

[21] Avila-Nava A Pech-Aguilar AG Lugo R Medina-Vera I Guevara-Cruz M Gutiérrez-Solis AL. Oxidative stress biomarkers and their association with mortality among patients infected with SARS-CoV-2 in Mexico. Oxidative Med Cell Longev. (2022) 2022:1–8. doi: 10.1155/2022/1058813 PMC9210126 35746958

[22] Delgado-Roche L Mesta F. Oxidative stress as key player in severe acute respiratory syndrome coronavirus (SARS-CoV) infection. Arch Med Res. (2020) 51:384–7. doi: 10.1016/j.arcmed.2020.04.019, PMID: 32402576 PMC7190501

[23] Mehri F Rahbar AH Ghane ET Souri B Esfahani M. Changes in oxidative markers in COVID-19 patients. Arch Med Res. (2021) 52:843–9. doi: 10.1016/j.arcmed.2021.06.004, PMID: 34154831 PMC8180845

[24] Gadotti AC Lipinski AL Vasconcellos FT Marqueze LF Cunha EB Campos AC. Susceptibility of the patients infected with Sars-Cov2 to oxidative stress and possible interplay with severity of the disease. Free Radic Biol Med. (2021) 165:184–90. doi: 10.1016/j.freeradbiomed.2021.01.044, PMID: 33524532 PMC7846460

[25] Spencer E Rosengrave P Williman J Shaw G Carr AC. Circulating protein carbonyls are specifically elevated in critically ill patients with pneumonia relative to other sources of sepsis. Free Radic Biol Med. (2022) 179:208–12. doi: 10.1016/j.freeradbiomed.2021.11.029, PMID: 34818575

[26] Jain SK Parsanathan R Levine SN Bocchini JA Holick MF Vanchiere JA. The potential link between inherited G6PD deficiency, oxidative stress, and vitamin D deficiency and the racial inequities in mortality associated with COVID-19. Free Radic Biol Med. (2020) 161:84–91. doi: 10.1016/j.freeradbiomed.2020.10.002, PMID: 33038530 PMC7539020

[27] Anwar S Almatroudi A Allemailem KS Jacob Joseph R Khan AA Rahmani AH. Protective effects of ginger extract against Glycation and oxidative stress-induced health complications: an in vitro study. PRO. (2020) 8:468. doi: 10.3390/pr8040468

[28] Mrityunjaya M Pavithra V Neelam R Janhavi P Halami PM Ravindra PV. Immune-boosting, antioxidant and anti-inflammatory food supplements targeting pathogenesis of COVID-19. Front Immunol. (2020) 11:570122. doi: 10.3389/fimmu.2020.570122, PMID: 33117359 PMC7575721

[29] Suhail S Zajac J Fossum C Lowater H McCracken C Severson N. Role of oxidative stress on SARS-CoV (SARS) and SARS-CoV-2 (COVID-19) infection: a review. Protein J. (2020) 39:644–56. doi: 10.1007/s10930-020-09935-8, PMID: 33106987 PMC7587547

[30] Loffredo L Violi F. COVID-19 and cardiovascular injury: a role for oxidative stress and antioxidant treatment? Int J Cardiol. (2020) 312:136. doi: 10.1016/j.ijcard.2020.04.066, PMID: 32505331 PMC7833193

[31] de Las HN Martín Giménez VM Ferder L Manucha W Lahera V. Implications of oxidative stress and potential role of mitochondrial dysfunction in COVID-19: therapeutic effects of vitamin D. Antioxidants (Basel). (2020) 9:897. doi: 10.3390/antiox9090897 32967329 PMC7555731

[32] De Flora S Balansky R La Maestra S. Rationale for the use of N-acetylcysteine in both prevention and adjuvant therapy of COVID-19. FASEB J. (2020) 34:13185–93. doi: 10.1096/fj.202001807, PMID: 32780893 PMC7436914

[33] Martín Giménez VM Inserra F Tajer CD Mariani J Ferder L Reiter RJ. Lungs as target of COVID-19 infection: protective common molecular mechanisms of vitamin D and melatonin as a new potential synergistic treatment. Life Sci. (2020) 254:117808. doi: 10.1016/j.lfs.2020.117808, PMID: 32422305 PMC7227533

[34] Beltrán-García J Osca-Verdegal R Pallardó FV Ferreres J Rodríguez M Mulet S. Oxidative stress and inflammation in COVID-19-associated sepsis: the potential role of anti-oxidant therapy in avoiding disease progression. Antioxidants (Basel). (2020) 9:936. doi: 10.3390/antiox9100936, PMID: 33003552 PMC7599810

[35] Ntyonga-Pono MP. COVID-19 infection and oxidative stress: an under-explored approach for prevention and treatment? Pan Afr Med J. (2020) 35:12. doi: 10.11604/pamj.2020.35.2.22877, PMID: 32528623 PMC7266475

[36] Polonikov A. Endogenous deficiency of glutathione as the Most likely cause of serious manifestations and death in COVID-19 patients. ACS Infect Dis. (2020) 6:1558–62. doi: 10.1021/acsinfecdis.0c00288, PMID: 32463221

[37] Qin M Cao Z Wen J Yu Q Liu C Wang F. An antioxidant enzyme therapeutic for COVID-19. Adv Mater. (2020) 32:e2004901. doi: 10.1002/adma.202004901 32924219

[38] Trujillo-Mayol I Guerra-Valle M Casas-Forero N Sobral MMC Viegas O Alarcón-Enos J. Western dietary pattern antioxidant intakes and oxidative stress: importance during the SARS-CoV-2/COVID-19 pandemic. Adv Nutr. (2021) 12:670–81. doi: 10.1093/advances/nmaa171, PMID: 33439972 PMC7929475

[39] Soto ME Guarner-Lans V Soria-Castro E Manzano Pech L Pérez-Torres I. Is antioxidant therapy a useful complementary measure for Covid-19 treatment? An algorithm for its application. Medicina (Kaunas). (2020) 56:386. doi: 10.3390/medicina56080386, PMID: 32752010 PMC7466376

[40] Shneider A Kudriavtsev A Vakhrusheva A. Can melatonin reduce the severity of COVID-19 pandemic? Int Rev Immunol. (2020) 39:153–62. doi: 10.1080/08830185.2020.1756284 32347747

[41] Poe FL Corn J. N-Acetylcysteine: a potential therapeutic agent for SARS-CoV-2. Med Hypotheses. (2020) 143:109862. doi: 10.1016/j.mehy.2020.109862, PMID: 32504923 PMC7261085

[42] El-Missiry MA El-Missiry ZMA Othman AI. Melatonin is a potential adjuvant to improve clinical outcomes in individuals with obesity and diabetes with coexistence of Covid-19. Eur J Pharmacol. (2020) 882:173329. doi: 10.1016/j.ejphar.2020.173329, PMID: 32615182 PMC7324339

[43] Lammi C Arnoldi A. Food-derived antioxidants and COVID-19. J Food Biochem. (2021) 45:e13557. doi: 10.1111/jfbc.13557 33171544

[44] Jorge-Aarón RM Rosa-Ester MP. N-acetylcysteine as a potential treatment for COVID-19. Future Microbiol. (2020) 15:959–62. doi: 10.2217/fmb-2020-0074, PMID: 32662664 PMC7359418

[45] Wang JZ Zhang RY Bai J. An anti-oxidative therapy for ameliorating cardiac injuries of critically ill COVID-19-infected patients. Int J Cardiol. (2020) 312:137–8. doi: 10.1016/j.ijcard.2020.04.009, PMID: 32321655 PMC7133895

[46] Guloyan V Oganesian B Baghdasaryan N Yeh C Singh M Guilford F. Glutathione supplementation as an adjunctive therapy in COVID-19. Antioxidants (Basel). (2020) 9:914. doi: 10.3390/antiox9100914, PMID: 32992775 PMC7601802

[47] Sies H Parnham MJ. Potential therapeutic use of ebselen for COVID-19 and other respiratory viral infections. Free Radic Biol Med. (2020) 156:107–12. doi: 10.1016/j.freeradbiomed.2020.06.032, PMID: 32598985 PMC7319625

[48] Xie X Zhong Z Zhao W Zheng C Wang F Liu J. Chest CT for typical coronavirus disease 2019 (COVID-19) pneumonia: relationship to negative RT-PCR testing. Radiology. (2020) 296:E41–5. doi: 10.1148/radiol.2020200343 32049601 PMC7233363

[49] Luraschi R Barrera-Avalos C Vallejos-Vidal E Alarcón J Mella-Torres A Hernández F. The comparative analysis of two RT-qPCR kits for detecting SARS-CoV-2 reveals a higher risk of false-negative diagnosis in samples with high quantification cycles for viral and internal genes. Can J Infect Dis Med Microbiol. (2022) 2022:1–10. doi: 10.1155/2022/2594564 PMC9259548 35812012

